# Role of NR4A family members in myeloid cells and leukemia

**DOI:** 10.1016/j.crimmu.2022.02.001

**Published:** 2022-02-22

**Authors:** Salix Boulet, Laure Le Corre, Livia Odagiu, Nathalie Labrecque

**Affiliations:** a Maisonneuve-Rosemont Hospital Research Center, Montréal, QC H1T 2M4, Canada; b Département de Microbiologie, Infectiologie et Immunologie, Université de Montréal, Montréal, QC H3C 3J7, Canada; c Département de Médecine, Université de Montréal, Montréal, QC H3C 3J7, Canada

**Keywords:** Nuclear receptors, NR4A1, NR4A2, NR4A3, Dendritic cells, Monocytes, Macrophages, Myeloid leukemia

## Abstract

The myeloid cellular compartment comprises monocytes, dendritic cells (DCs), macrophages and granulocytes. As diverse as this group of cells may be, they are all an important part of the innate immune system and are therefore linked by the necessity to be acutely sensitive to their environment and to rapidly and appropriately respond to any changes that may occur. The nuclear orphan receptors NR4A1, NR4A2 and NR4A3 are encoded by immediate early genes as their expression is rapidly induced in response to various signals. It is perhaps because of this characteristic that this family of transcription factors has many known roles in myeloid cells. In this review, we will regroup and discuss the diverse roles NR4As have in different myeloid cell subsets, including in differentiation, migration, activation, and metabolism. We will also highlight the importance these molecules have in the development of myeloid leukemia.

## Highlights

• NR4A1-3 have important roles in the different cells of the myeloid compartment.
• These orphan receptors homeostasis, differentiation, and activation.
• NR4A family is important in suppressing the development of myeloid leukemias.
• NR4As have been linked to several diseases and could be pharmacological targets.

## Graphical abstract

**Figure:**
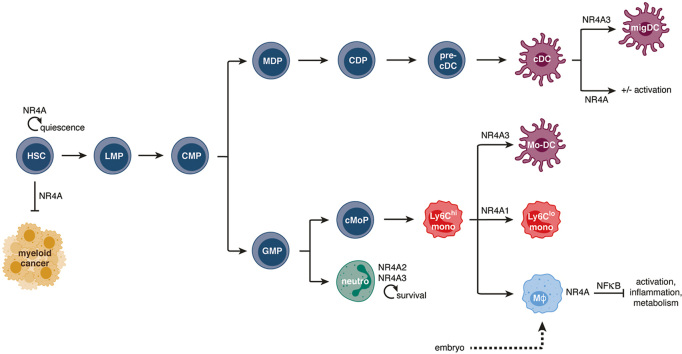

## Abbreviations

AF: activation function
AML: acute myeloid leukemia
cDC: conventional DC
CDP: common DC progenitor
cMoP: common monocyte progenitor
CoREST: co-repressor for RE1 silencing transcription factor
DC: dendritic cells
DHE: dihydroergotamine
FPD: familial platelet disorder
HSC: Hematopoietic stem cells
LDL: low-density lipoprotein
MDS: myelodysplastic syndrome
Mo-DC: monocyte-derived DC
MPN: myeloproliferative neoplasm
NE: norepinephrine
PD: Parkinson's disease
pDC: plasmacytoid DC
PDNPA: 2-(3,5-dihydroxy-2-nonanoylphenyl)acetate
TSS: transcription start site

## 1 Introduction

**Fig. 1:**
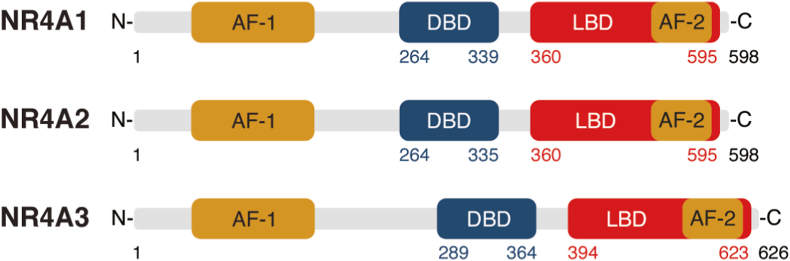
Structure of NR4A nuclear receptors. Schematic representation of NR4A1 (Nur77), NR4A2 (Nurr1) and NR4A3 (Nor-1) with their most important functional domains. These include an N-terminal activation function (AF-1) domain and a C-terminal AF-2 domain surrounding the DNA-binding domain (DBD) and the ligand-binding domain (LBD). Numbers indicate amino acid positions of human proteins.

Nuclear receptors (NRs) are a family of structurally similar proteins specialized in the conversion of environmental signals into complex cellular responses, mainly through their action as transcription factors. They are important in a wide range of physiological processes including development, metabolism or reproduction and their clinical importance is illustrated by the fact that this family of proteins is highly targeted by prescription drugs (Overington et al., 2006). There are 49 NRs in mice and 48 in humans (Chambon, 2005). Structurally, NRs have a highly conserved central zinc-finger containing a DNA-binding domain, a C-terminal ligand-binding domain and a variable amino-terminal region, which contains an activation function (AF-1) domain that mediates protein-protein interactions (Fig. 1). In addition to its ligand-binding function, the C-terminal domain is involved in transcriptional co-regulator interaction through conformational changes of the AF-2 domain mediated by ligand-binding (Gronemeyer et al., 2004; Sever and Glass, 2013).

While most NRs have known ligands, several have no identified ligands and are thus termed ‘orphan’ receptors. These comprise the oldest evolutionary members of the family and homologs can be found in invertebrate species. In drosophila, for example, most NRs are orphans (Fahrbach et al., 2012). Because they have no known ligands, the function of orphan NRs could instead be modulated by their expression pattern or by the regulation of the accessibility of their DNA binding sites in different cells or cell states (Evans and Mangelsdorf, 2014).

NR4A family members of nuclear receptors, NR4A1, NR4A2 and NR4A3 (see Fig. 1 - also known as Nur77, Nurr1 and NOR-1, respectively) are rapidly induced in response to a number of stimuli, including T-cell receptor signalling in T cells, G-protein coupled receptors, mechanical stress, protein kinase receptors and cyclic AMP activation (Moran et al., 2011; Pearen and Muscat, 2010) and are known as immediate early genes because their expression is usually induced within minutes of stimulation (Pearen and Muscat, 2010). Crystallographic studies of NR4A2, NR4A1 and of the *Drosophila* ortholog DHR38 have shown that the ligand-binding pocket of these proteins is filled by hydrophobic residues, providing a structural argument for the fact that NR4As would be orphan receptors (Baker et al., 2003; Flaig et al., 2005; Wang et al., 2003). However, recent studies have revealed that the NR4A2 ligand-binding domain is highly dynamic and may directly bind molecules such as 5,6-dihydroxyindole, prostaglandins E1 and A1 (Bruning et al., 2019; Jang et al., 2021; Rajan et al., 2020; Safe et al., 2016; Vera et al., 2019). This suggests that endogenous ligands may yet be uncovered for the whole NR4A family. As most NRs, NR4A1, -2 and -3 can act as transcription factors, recognizing the NBRE motif (nerve growth factor-induced protein B-responsive element: AAAGGTCA) on DNA as monomers or the NurRE palindromic motif (TGATATTTX_6_AAATGCCA) as homodimers (Philips et al., 1997; Wilson et al., 1991; Winoto and Littman, 2002). They also have known non-nuclear roles, such as converting the anti-apoptotic molecule Bcl2 to its pro-apoptotic Bcl2-BH3 form in the mitochondria (Li et al., 2000; Lin et al., 2004; Thompson and Winoto, 2008).

**Fig. 2:**
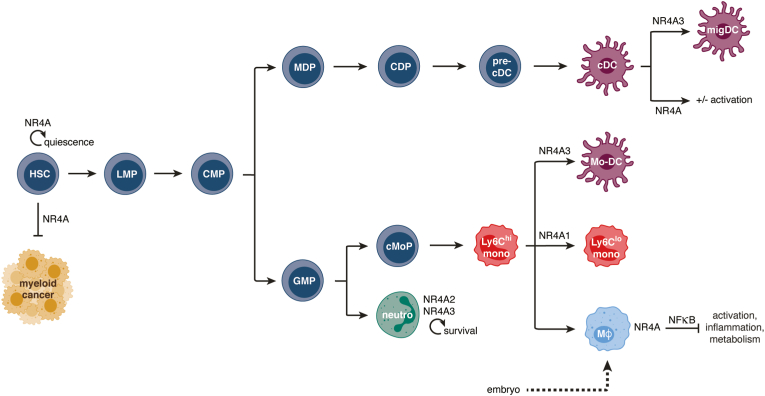
Overview of the roles of NR4A family members in myeloid cells. The schematic depicts the different roles NR4As have on the myeloid lineage, in progenitors, monocytes, macrophages, DCs and neutrophils. NR4As have been shown to regulate progenitor quiescence and limit myeloid leukemia. In DCs, NR4As can modulate activation, while only NR4A3 is required for proper migration from peripheral tissues to draining lymph nodes. NR4A1 is specifically required for differentiation of Ly6C^hi^ to Ly6C^lo^ monocytes and NR4A3 for the differentiation of Mo-DCs induced in response to microbial stimulation. NR4As regulate macrophage activation and inflammatory response, and their metabolism. Finally, NR4A2 and NR4A3 have been shown to positively regulate neutrophil survival.

Myeloid cells are a group of innate leukocytes that include monocytes, macrophages, dendritic cells (DCs) and granulocytes. While they have diverse functions, they are usually known to be rapid responders in case of infection or tissue injury and are acutely sensitive to changes in the microenvironment. Because of their regulation and response patterns, NR4As are perfectly designed to be sensors of environmental changes. It is thus not surprising that several important roles for NR4As in myeloid cell biology have been uncovered over the past years, from proper myeloid cell development to differentiation and activation (Fig. 2). In this review, we propose to cover these different aspects of the roles of NR4As.

## 2 The non-redundant role of NR4As in monocyte differentiation

Early studies on the role of NR4As in T cells highlighted the redundancy of these molecules in regulating apoptosis of thymocytes (Winoto and Littman, 2002). Given their similar expression pattern and the homology of their DNA binding domain, this was not surprising. However, increasing evidence shows that these molecules can have distinct functions depending on the cellular context, and this is perhaps best illustrated by the role of NR4A1 and NR4A3 in the biology of monocytes. Monocytes are circulating cells that can be divided in humans into three populations based on the expression of CD14 and CD16 (Passlick et al., 1989): CD14^+^CD16^−^, CD14^+^CD16^+^ and CD14^low^CD16^+^ cells. In mice, monocytes are divided largely as a function of Ly6C expression into Ly6C^hi^CX3CR1^int^CCR2^+^CD62L^+^CD43^low^ and Ly6C^lo^CX3CR1^hi^CCR2^low^CD62L^−^CD43^+^ cells while Ly6C intermediate cells are also found (Geissmann et al., 2003; Jakubzick et al., 2013; Jung et al., 2000). Transcriptional analyses of mouse and human monocytes have established a strong correlation of CD14^+^CD16^−^ with murine Ly6C^hi^ cells and CD14^low^CD16^+^ with Ly6C^lo^ monocytes (Ingersoll et al., 2010). While these monocyte subpopulations are still in use and are experimentally convenient, single-cell RNA sequencing experiments have demonstrated further heterogeneity in mouse and human monocytes (Mildner et al., 2017; Villani et al., 2017).

Ly6C^hi^ monocytes are commonly referred to as classical monocytes. These are descendants of the common monocyte progenitor (cMoP). They circulate in the blood and can extravasate under homeostatic conditions into tissues. Non-classical Ly6C^lo^ monocytes patrol the vasculature via LFA/ICAM dependent crawling on resting endothelium (Auffray et al., 2007; Carlin et al., 2013). Similar to Ly6C^hi^ monocytes, non-classical monocytes can be recruited to sites of inflammation of tissue damage. Human CD14^low^CD16^hi^ monocytes also display crawling behaviour once transferred into *Rag2*^*−/−*^*Il2rg*^*−/−*^*Cx3cr1*^*gfp*^ immunocompromised mice (Cros et al., 2010). Several labelling, depletion, or transfer experiments in both human and animal models have demonstrated that non-classical monocytes derive from classical monocytes (Mildner et al., 2017; Patel et al., 2017; Sunderkötter et al., 2004; Tacke et al., 2006; Varol et al., 2007; Yona et al., 2013).

### 2.1 NR4A1 regulates the differentiation of non-classical monocytes

**Fig. 3:**
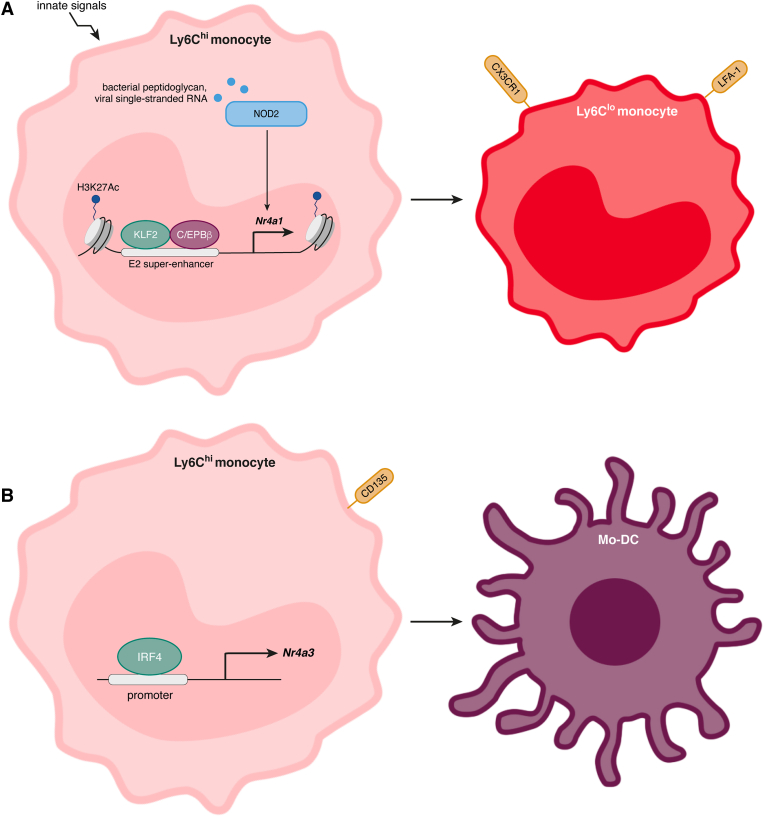
NR4A1 and NR4A3 have different roles in monocytes. (A) Stimuli, such as microbial stimulation, can activate innate receptors (e.g. NOD2) and induce the expression of *Nr4a1* in monocytes. This expression, which is regulated by binding of C/EBPβ and KLF2 to an enhancer region (E2) of *Nr4a1*, is required for the proper differentiation of Ly6C^hi^ into Ly6C^lo^ monocytes. In absence of NR4A1, Ly6C^lo^ monocytes that do survive fail to properly express CX3CR1 and LFA-1. (B) Part of the transcriptional program induced by IRF4 during the differentiation of Ly6C^hi^ monocytes into Mo-DCs results from the direct activation of NR4A3. A subset of Ly6C^hi^ monocytes, CD135^+^ monocytes, are the likely progenitors in this differentiation process.

The conversion of murine classical to non-classical monocytes is accompanied by an increase in the expression of NR4A1, but not of the other members of its family (Hanna et al., 2011; Mildner et al., 2017). This is consistent with the fact that NR4A1 is required for the generation of Ly6C^lo^ monocytes (Fig. 3). Indeed, *Nr4a1*^*−/−*^ mice have a dramatic deficiency in these cells, with only 5–10% of the population remaining (Carlin et al., 2013; Hanna et al., 2011). In absence of NR4A1, Ly6C^lo^ monocytes have an increase in DNA damage, abnormally progress through the cell cycle and eventually die (Hanna et al., 2011). Those Ly6C^lo^ monocytes that are found in *Nr4a1*^*−/−*^ deficient mice have largely reduced patrolling behaviour and decreased CX3CR1 and LFA-1 expression, molecules important for cell migration (Hanna et al., 2011). Whether NR4A1 plays a similar role in the generation of human non-classical monocytes requires further investigation, but single-cell RNA sequencing of human peripheral blood has shown an increase in *NR4A1* transcription in CD14^dim^CD16^high^ monocytes (Villani et al., 2017). In addition, the molecular targets through which NR4A1 regulates the differentiation of non-classical monocytes are still not clearly defined.

Epigenetic studies of enhancer usage by ChIP-seq of chromatin marks in murine MDPs, cMoPs, Ly6C^hi^ and Ly6C^lo^ monocytes support the lineage relationship of Ly6C^hi^ and Ly6C^lo^ monocytes (Mildner et al., 2017; Thomas et al., 2016). *Nr4a1* is amongst the genes where there is a selective and robust acquisition of active epigenetic marks, such as H3K27Ac, in the transition from Ly6C^hi^ to Ly6C^lo^ (Mildner et al., 2017; Thomas et al., 2016). Consistent with its role in lineage specification and function, *Nr4a1* expression in Ly6C^lo^ monocytes is regulated by a super-enhancer, a molecular structure that is usually specific to cell types or subtypes (Thomas et al., 2016). Targeted CRISPR-*Cas9* deletion of a subdomain of this super-enhancer (termed E2), results in a loss of Ly6C^lo^ monocytes, duplicating the results obtained in *Nr4a1*^*−/−*^ mice. However, in E2-deficient mice, *Nr4a1* expression in other cell types is maintained and the LPS-induced upregulation of *Nr4a1* in peritoneal macrophages is unaffected, demonstrating that this enhancer activity is specific to non-classical monocytes (Thomas et al., 2016). Interestingly, the orthologous region to E2 in humans also had enriched H3K27Ac chromatin marks in CD14^dim^CD16^+^ non-classical monocytes when compared to CD14^+^CD16^−^ monocytes, suggesting similar regulation of *NR4A3* in humans. KLF2 and C/EBPβ are transcription factors that bind to this enhancer region and regulate the expression of *Nr4a1*, which makes these molecules also required for the development of Ly6C^lo^ monocytes (Fig. 3) (Guilliams et al., 2018; Mildner et al., 2017; Thomas et al., 2016).

The signals that favor the transition of monocytes from classical monocytes to patrolling monocytes are largely unknown. The NOD2 receptor recognizes fragments of bacterial peptidoglycan to stimulate the innate arm of the immune system (Girardin et al., 2003; Grimes et al., 2012). It has also been reported to recognize viral single-stranded RNAs (Sabbah et al., 2009). NOD2 is expressed by monocytes and *in vivo* injection of a NOD2 agonist in mice induces expression of transcripts that encode for C/EBPβ and NR4A1 in Ly6C^hi^ monocytes (Lessard et al., 2017). This expression profile is accompanied by an increase in the proportion of circulating Ly6C^lo^ monocytes, a phenomenon that was not observed in NOD2^−/−^ mice and was duplicated on freshly isolated human blood monocytes treated *ex vivo* with the same NOD2 agonist(Lessard et al., 2017). These observations suggest that innate receptor signalling could be a mediator of both murine and human monocyte differentiation through the induction of NR4A1 expression (Fig. 3).

Our understanding of the biological importance of Ly6C^lo^ monocytes has progressed since the discovery that NR4A1 is required for their development (Hanna et al., 2011). Different analyses have shown that one of the important roles of non-classical monocytes is to remove unwanted cells or molecules. In response to intravenous injection of cancer cells in mice, Ly6C^lo^ monocytes rapidly extravasate to tumor sites and engulf tumorous material (Hanna et al., 2015). Importantly, *in vitro* assays confirmed that human CD14^low^CD16^+^ monocytes similarly engulf tumor material (Hanna et al., 2015). Consistent with these observations, a protective role for Ly6C^lo^ monocytes has been observed in both induced and spontaneous models of metastatic lung cancer, where *Nr4a1*^*−/−*^ mice have increased metastatic lesions (Hanna et al., 2015). Interestingly, adoptive transfer of wild-type Ly6C^lo^ but not Ly6C^hi^ monocytes partially protected *Nr4a1*^*−/−*^ mice from lung tumor metastasis following intravenous injection of B16F10 melanoma (Hanna et al., 2015). Another such example of the role of Ly6C^lo^ monocytes comes from the study of Alzheimer's disease. Accumulation of amyloid-β is a hallmark of this disease. In the APP/PS1 (amyloid precursor protein/presenilin 1) transgenic mouse model of Alzheimer's, Ly6C^lo^ monocytes were shown to crawl to veins harbouring amyloid-β and internalize these aggregates (Blanchard et al., 2003; Michaud et al., 2013). Grafting myeloablated APP/PS1 mice with *Nr4a1*^*−/−*^ bone marrow cells resulted in increases in the number and area of amyloid-β deposits when compared to mice reconstituted with WT cells. This suggests a possible role for Ly6C^lo^ monocytes in restraining Alzheimer's. Thus, in models for Alzheimer's and metastatic lung cancer, patrolling monocytes have protective functions. However, they can also have deleterious effects. In murine single-lung transplants, donor-derived Ly6C^lo^ monocytes produce CXCL12 which recruits neutrophils. These cells can cause tissue damage and primary graft dysfunction, a process that is largely reduced if the donor graft is from a NR4A1-deficient mouse or if it is pre-treated with clodronate-liposomes and therefore depleted of Ly6C^lo^ monocytes, suggesting possible future pharmacological treatment for patients (Zheng et al., 2017). Therefore, the knowledge that NR4A1 is required for the differentiation of non-classical monocytes has led to several studies aimed at understanding the biological importance of this cell subset.

### 2.2 NR4A3 regulates the differentiation of monocytes into DCs

At steady-state, no monocyte progenitor or mature populations are affected in NR4A3-deficient mice (Boulet et al., 2019). However, upon the culture of monocytes with GM-CSF and IL-4, a common method for generating DCs *in vitro* (Inaba et al., 1992; Sallusto and Lanzavecchia, 1994), *Nr4a3* expression is strongly induced both in murine and human cells (Boulet et al., 2019; Briseño et al., 2016; Lehtonen et al., 2007). Studies have shown that NR4A3 is required for the proper differentiation of monocytes into DCs in these cultures, NR4A3-deficiency resulting in an up to a 10-fold reduction in the number of DCs generated (Boulet et al., 2019; Park et al., 2016). The *in vivo* equivalent of GM-CSF + IL-4 differentiated monocytes is still debated, but it has been proposed to be a population of LPS-induced DC-SIGN^+^ monocyte-derived DCs (Mo-DCs) found in skin draining lymph nodes of mice (Cheong et al., 2010). These cells could originate specifically from a CD135-expressing Ly6C^hi^ monocytes subset (Boulet et al., 2019; Menezes et al., 2016). Interestingly, DC-SIGN^+^ Mo-DCs are largely absent in LPS-injected NR4A3-deficient mice, which results in a decrease in the priming of CD8 T cell responses to gram-negative bacteria (Boulet et al., 2019). Transcriptomic analysis of murine WT and *Nr4a3*^*−/−*^ monocytes during *in vitro* Mo-DC differentiation revealed that this nuclear receptor regulates the balance of DC- and macrophage-defining genes (Boulet et al., 2019). Indeed, *Nr4a3*^*−/−*^ cells cultured with GM-CSF + IL-4 express more macrophage-defining receptors such as CD64, F4/80 and Mertk and are less efficient at antigen presentation than their WT counterparts (Boulet et al., 2019). In addition, these studies have shown that NR4A3 is a direct effector of the IRF4-mediated program required for the differentiation of monocytes into Mo-DCs (Fig. 3), and overexpression of NR4A3 in IRF4-deficient cells partially rescues *in vitro* Mo-DC generation (Boulet et al., 2019; Briseño et al., 2016). Illustrating the fact that NR4A1 is not involved in this process and is perhaps even deleterious, is that NUR77-GFP^+^ monocytes cannot differentiate *in vitro* into DCs (Briseño et al., 2016). Furthermore, *Nr4a1*^*−/−*^ monocytes do not have an *in vitro* defect of differentiation into Mo-DCs (Briseño et al., 2016).

NR4A1 and NR4A3 have therefore distinct roles in monocyte function, while no clear role has been uncovered for NR4A2. NR4A3 is not required for the transition of classical monocytes to non-classical monocytes (Boulet et al., 2019). The roles these molecules play in monocyte biology are thus non-redundant and perhaps even in opposition given that NR4A1-expressing Ly6C^lo^ monocytes cultured with GM-CSF and IL-4 are virtually incapable of differentiating into Mo-DCs (Briseño et al., 2016). Understanding this dichotomy could provide insight into the molecular uniqueness of these homologous transcription factors and it might be interesting to explore whether a super-enhancer domain in the context of Mo-DC differentiation regulates *Nr4a3* expression, as one does for *Nr4a1* during non-classical monocyte differentiation. Given the different biological roles of Ly6C^hi^ or Ly6C^lo^ monocytes and Mo-DCs in contexts of infection, cancer, or transplantation, targeting their balance could reveal itself as an important therapeutic approach. The further study of NR4As would provide part of the answers to this question.

## 3 DC activation and NR4As

DCs are highly efficient antigen-presenting cells that have crucial roles in initiating adaptive immunity and maintaining tolerance. Due to their biological importance and their potential as therapeutic targets, the molecular processes that regulate their development, activation and function have been under active investigation. DCs differentiate from a committed bone marrow progenitor, the common DC progenitor (CDP). This progenitor can differentiate into pre-DCs, which eventually give rise to conventional DCs (cDCs). These cDCs, which can be further divided into cDC1 or cDC2, are distinct in their ontogeny and molecular requirements from Mo-DCs, derived from monocytes. Gene expression analysis of murine DC progenitors and mature DCs has shown that expression of transcripts coding for the three NR4A receptors increases at the transition from pre-DC to mature DC (Grajales-Reyes et al., 2015). However, there is no evidence that NR4A1 and NR4A3 are required for the development of DCs, as these are properly generated in deficient mice (Boulet et al., 2019; Park et al., 2016; Tel-Karthaus et al., 2018). *Nr4a2* is the least expressed of the family in DCs, while *Nr4a1* is more strongly expressed in resident lymphoid-tissue DCs (resident DCs) and in plasmacytoid DCs, a DC subset with a distinct ontogeny (Dress et al., 2020; Karthaus et al., 2013; Park et al., 2016; Tel-Karthaus et al., 2018). There is, however, no known role for NR4A1 in the homeostasis of these cells (Park et al., 2016; Tel-Karthaus et al., 2018).

### 3.1 NR4A3 and DC migration

*Nr4a3* expression is enriched in DCs that have migrated from the tissues to draining lymph nodes (migratory DCs) (Park et al., 2016). Consistent with this expression pattern, migratory DCs specifically require NR4A3, and deficient mice have a dramatic decrease in this process (Boulet et al., 2019; Park et al., 2016). The migration of lymphoid and dendritic cells is highly dependent on the chemokine receptor CCR7 (Förster et al., 1999; Ohl et al., 2004; Stein et al., 2000). As NR4A3-deficient DCs express low levels of CCR7 at steady-state and upon activation (Boulet et al., 2019; Park et al., 2016), this is likely the mechanism by which NR4A3 regulates DC migration. Interestingly, defective CCR7 expression is regulated by NR4A3 specifically in DCs, as T cells and B cells in deficient mice had normal levels of this molecule (Park et al., 2016). Direct binding of NR4A3 to the *Ccr7* promoter was not detected and regulation is probably indirect, perhaps through the *Ccr7*-regulating transcription factor FOXO1 (Park et al., 2016). It is also possible that NR4A3 regulates a broader migratory transcriptional program, beyond the expression of *Ccr7*. Indeed, migration of DCs is associated with a unique gene signature that includes genes that favor cell motility and migration like *Ccr7,* but also *Arc*, *Fscn1*, *Actn1* (Miller et al., 2012; Ohl et al., 2004; Stanley et al., 2008; Ufer et al., 2016; Yamakita et al., 2011). A large portion of this migratory signature is regulated by NR4A3 in *in vitro* generated Mo-DCs (Boulet et al., 2019). The deficiency of migratory cDCs observed in *Nr4a3*^*−/−*^ mice may also be the consequence of defective regulation of this NR4A3-dependent migratory signature, further studies should reveal whether this is the case. Additionally, given that NR4As intersect with the NFκB pathway in other myeloid cells (see below), NR4A3 may be required for the proper induction of the NFκB-regulated migratory DC gene network (Baratin et al., 2015), which includes *Ccr7*. However, this has not been explored.

### 3.2 The NR4A family in the process of DC activation

DCs are a relatively rare cell type, thus research attempting to uncover the role of NR4As in the response of these cells has often been done *in vitro*. Two main culture protocols are used to generate DCs. Historically, culturing bone marrow cells with GM-CSF with or without the addition of IL-4 has been the first and possibly most widely used method (Inaba et al., 1992; Lu et al., 1995). However, it has been clear for decades that the product of this culture is heterogeneous and that both macrophage- and DC-like cells are obtained (Helft et al., 2015; Inaba et al., 1992; Menges et al., 2005). More recently, it was shown that different progenitors, including CDPs, cMoPs and monocytes contribute to the final culture (Helft et al., 2015). Therefore, conclusions made on the molecular processes that govern different aspects of DC development, activation and function in these conditions are not necessarily straightforward. This is particularly true for studies looking at the role of NR4A3 in DC biology, given that NR4A3-deficiency affects the differentiation of progenitors in response to GM-CSF (Boulet et al., 2019). Conversely, bone marrow cells can be cultured with FMS-like tyrosine kinase 3 ligand (FLT3L). This generates cDCs and pDCs and is thought to reflect more accurately the physiologic development of DCs (Naik et al., 2005). The end-product of both GM-CSF and FTL3L DC cultures has been used to study the role of NR4As in DC activation, which follows from stimulation with inflammatory mediators or with conserved pathogen molecular patterns and results in DCs upregulating the expression of molecules required to stimulate an immune response (Sousa, 2004). Across all culture models surveyed, *Nr4a3* is systematically upregulated upon DC activation with viral infection or TLR stimulation (Karthaus et al., 2013; Nagaoka et al., 2017; Ng et al., 2011; Wang et al., 2009). Upon activation of murine DCs generated using the FLT3L-differentiation protocol, NR4A3 can modulate the activity of IRF3 and IRF7 on the promoter of IFNβ (Ng et al., 2011). In GM–CSF–generated DCs, limiting *Nr4a3* expression by siRNA in murine DCs prior to stimulation with LPS, CpG or polyI:C results in suboptimal upregulation of co-stimulatory molecules CD80 and CD86 as well as reduced *Il6*, *Il12b* and *Il10* transcription (Nagaoka et al., 2017). IRF4, IRF8 and IKKβ are all decreased in absence of NR4A3 and knock-down experiments for each of these at least partially recapitulate the NR4A3-deficient phenotype, suggesting that these may be important molecular targets in this experimental setup.

Data for NR4A1 and NR4A2 is more contradictory. Two different studies show that *Nr4a1* is first up-regulated and then down-regulated in response to TLR stimulation (Karthaus et al., 2013; Tel-Karthaus et al., 2018). Another study has shown that upon infection with murine cytomegalovirus or Newcastle Disease virus, FLT3L-generated DCs downregulate the expression of *Nr4a1* and *Nr4a2* (Ng et al., 2011). Finally, Saini *et al.* demonstrated in GM-CSF cultures that *Nr4a2* is induced in response to LPS stimulation (Saini et al., 2016). These discrepancies might arise from experimental differences such as *in vitro* DC models, type of stimulation or activation time. For example, the expression pattern of *Nr4a1* and *Nr4a2* in response to viral infection could be influenced by an evasion mechanism. Regardless of whether their expression is induced or repressed, these molecules are responsive to TLR stimulation, and they seem important in properly tuning DC activation. Indeed, limiting *Nr4a1* expression in murine FLT3L DCs and human *in vitro*-generated Mo-DCs increased their production of IL-6, TNFα and/or IL-12 in response to TLR stimulation while *Nr4a2* overexpression in murine GM-CSF + IL-4 cultures restrained IL-12 and TNFα production while increasing IL-10 production (Saini et al., 2016; Tel-Karthaus et al., 2018). This suggests that NR4A1 and NR4A2 broadly act to limit the inflammatory response of *in vitro* generated DCs upon activation, serving as a molecular brake on the immune system to mitigate the possible negative effects of an overzealous response.

With increasingly improved knowledge on DC development, subpopulations, and tissue specificities, it is perhaps time to re-explore the role of NR4As in the biology of this cell population. It would be of interest to dissect out the unique and overlapping roles of each NR4A molecule as well as to better characterize, using modern techniques such as single-cell RNA sequencing and ATAC-seq, the molecular roles and the molecular targets of NR4A1, 2 and 3 in the response of cDCs to an immunological challenge. It was recently shown that NR4A expression in tumor infiltrating lymphocytes has an impact on tumor control (Chen et al., 2019; Liu et al., 2019; Odagiu et al., 2020). It would therefore be worthwhile to study their function in the myeloid cells infiltrating the tumor as well.

## 4 NR4As modulate the inflammatory response of macrophages

While monocytes can differentiate into macrophages upon stimulation, the majority of tissue macrophages are of embryonic origin (Ginhoux and Jung, 2014; Sawai et al., 2016). Most evidence suggests that NR4As do not have a role in their development (Boulet et al., 2019; Park et al., 2016; Thomas et al., 2016). However, Tacke et al. uncovered a novel CD11b^−^F4/80^+^CD64^+^Tim4^+^ resident macrophage population in the thymus that required NR4A1 (Tacke et al., 2015). Apoptosis is common in the thymus, as many thymocytes fail to be successfully selected during their maturation process. Thymus CD11b^−^ resident macrophages are efficient at clearing apoptotic cells, restraining inflammation and damage caused by dying cells, and their absence in *Nr4a1*^*−/−*^ mice correlates with increased transcription of pro-inflammatory cytokines, accelerated thymic shrinkage (termed involution, a process that occurs with age) and increased production of anti-nuclear autoantibodies (Tacke et al., 2015). However, given that these observations were made in full *Nr4a1*^*−/−*^ mice, they could possibly result from the roles that NR4A1 has in regulatory T cell development or thymocyte negative selection (Odagiu et al., 2021). Otherwise, while the differentiation of classical monocytes into Ly6C^lo^ monocytes requires NR4A1, their differentiation into Ly6C^lo^ macrophages is not affected in NR4A1-deficient mice (Hilgendorf et al., 2014; Varga et al., 2013). Thus, the role of NR4As in macrophages is subtle at steady-state. It is upon activation with molecules whose presence would correlate with dramatic environmental changes *in vivo*, such as TLR ligands, that these receptors seem important.

### 4.1 NR4As limit the inflammatory macrophage response

In macrophages, NR4As are induced in response to several pro-inflammatory stimuli such as TLR stimulation, IFNγ, TNFα and oxidized low-density lipoprotein (LDL) (Barish et al., 2005; Bonta et al., 2006; Ipseiz et al., 2014; Mahajan et al., 2015; Pei et al., 2005; Shao et al., 2010). Given this expression profile, it is not surprising that this family of nuclear orphan receptors is important in diseases or models that have an inflammatory component such as Parkison's disease (PD), atherosclerosis and sepsis. As discussed below, the role of NR4As in macrophages in these different contexts is largely, although not exclusively, to regulate the activation of the NFκB pathway.

**Fig. 4:**
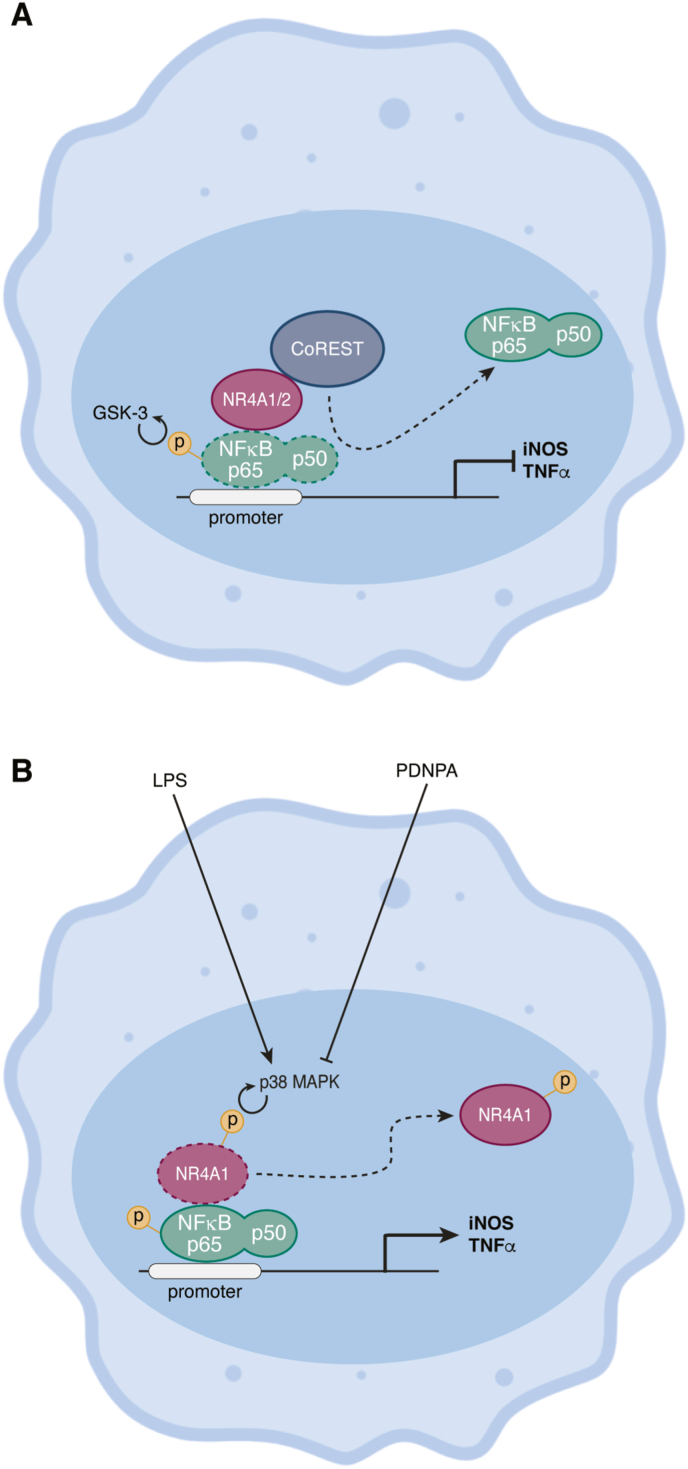
Molecular inhibition of the NFκB pathway by NR4As in macrophages/microglia. (A) GSK-3 phosphorylates the NFκB p65 subunit, which allows for docking of NR4A on NFκB bound to DNA promoter region. NR4A1 or NR4A2 is then capable of recruiting CoREST, which removes the NFκB subunits from their target DNA. This process, where NR4A acts as an intermediate to mediate transcriptional repression without directly binding to DNA is termed transrepression. (B) Interaction of NR4A1 with NFκB is inhibited by p38 MAPK phosphorylation. Once so phosphorylated, NR4A1 no longer inhibits the NFκB pathway and the inflammatory response is maintained. The chemical compound n-pentyl 2-(3,5-dihydroxy-2-nonanoylphenyl)acetate (PDNPA) inhibits the interaction between p38 and NR4A1 and could thus be used as an inhibitor of inflammation.

While the exact aetiology of PD is not completely understood, there is an inflammatory component as illustrated by the activation of central nervous system resident macrophages, termed microglia, and elevated levels of pro-inflammatory cytokines (Block et al., 2007; Nagatsu and Sawada, 2005). *Nr4a2* germline deletion in mice results in the reduction of dopaminergic neurons and human mutations that lead to decreased *Nr4a2* transcription are linked to late-onset familial PD (Le et al., 2003; Zetterström et al., 1997). This is highly suggestive of a protective role for NR4A2 in PD. Saijo et al. have shown that, in response to LPS, *Nr4a2* expression is induced in microglia and that its inhibition via substantia nigra injection of lentiviruses encoding for *Nr4a2*-specific shRNAs (which preferentially transduces microglia and astrocytes) causes an increase in regional levels for transcripts encoding the inflammatory mediators TNFα, iNOS and IL-1β and accentuates neuronal damage (Saijo et al., 2009). This is because, as demonstrated in the BV-2 microglial cell line and in RAW264.7 cells, NR4A2 mediates a negative feedback regulation of the NFκB pathway by transrepression, a process whereby the nuclear receptor does not bind directly to DNA but rather associates with it indirectly via interaction with a target transcription factor (Glass and Saijo, 2010; Saijo et al., 2009). Once it docks to the GSK3-phosphorylated p65 unit of NFκB, NR4A2 can recruit the CoREST (Co-repressor for RE1 silencing transcription factor) complex (Saijo et al., 2009). CoREST then actively removes p65 from promoters of inflammatory genes such as iNOS and TNFα leading to reduced transcription of these genes (Fig. 4).

NR4A1 can also recruit CoREST in macrophages (Shaked et al., 2015). Norepinephrine (NE) is involved in the pathology of experimental autoimmune encephalitis, the murine model for multiple sclerosis. NR4A1-deficiency was shown to cause elevated NE production and exacerbated disease, a result that was linked to the fact that, in the RAW macrophage cell line, NR4A1 knock-down decreased recruitment of CoREST at the promoter of tyrosine hydroxylase, the rate-limiting enzyme in catecholamine synthesis, which increased its transcription (Shaked et al., 2015). In addition, in LPS-treated peritoneal macrophages, electrophoretic mobility shift assays showed that NR4A1 disrupts p65-binding to NFκB DNA probes, although it is unknown if CoREST is an intermediary in this case as well (Li et al., 2015). Interestingly, NR4A1 interference of p65 was inhibited by phosphorylation of the former by LPS-induced MAPK p38α, thus releasing the NR4A1-mediated inhibition of the NFκB pathway and allowing for the induction of an inflammatory response (Fig. 4). Given that NR4A1 and p38α interaction can be inhibited by the chemical compound n-pentyl 2-(3,5-dihydroxy-2-nonanoylphenyl)acetate (PDNPA), injection of PDNPA prior to injection of LPS decreased the release of inflammatory mediators and improved mouse survival (Li et al., 2015). Because PDNPA had no effect on cytokine secretion from NR4A1-deficient macrophages or failed to rescue *Nr4a1*^*−/−*^ mice from septic shock, the effect of this chemical compound is not likely to be off-target (Li et al., 2015). This interaction between p38α and NR4A1 could be of interest for the development of novel anti-inflammatory agents.

Macrophages play key roles in atherosclerosis (Moore and Tabas, 2011). Modified LDLs that accumulate in the arterial vessel wall initiate an inflammatory response that leads to the recruitment of monocytes and their differentiation into macrophages. These then internalize the modified lipids via scavenger receptors such as CD36 and type A scavenger receptor and become foam cells, which can contribute to lesion progression by their pro-inflammatory nature or by necrosis. Both smooth muscle cells and macrophages in the atherosclerotic lesion express NR4As (Arkenbout et al., 2002; Bonta et al., 2006, 2010; Pei et al., 2005; Zhao et al., 2010). Crossing full *Nr4a3* knock-out mice to apolipoprotein E (Apoe) deficient mice, used to model atherosclerosis, resulted in decreased atherosclerotic lesions (Zhao et al., 2010). Interestingly, when the same group grafted *Apoe*^*−/−*^ mice with wild-type or *Nr4a3*^*−/−*^ bone marrow cells an increase in lesions was observed (Qing et al., 2014). These seemingly contradictory results are explained by the role NR4A3 has in the non-hematopoietic compartment. In human vascular endothelial cells, NR4A3 overexpression induced VCAM-1 and ICAM-1 expression. NR4A3-deficiency in endothelial cells could therefore modulate the adhesion of monocytes to the affected area (Zhao et al., 2010). This confounding factor was eliminated when using bone-marrow chimeras (Qing et al., 2014). Similarly, grafting NR4A1-deficient bone marrow into *Ldlr*^*−/−*^ mice resulted in an increase in atherosclerotic lesions (Hamers et al., 2012; Hanna et al., 2012; Koenis et al., 2018). Modulation of NR4A expression using vectors on bone-marrow-derived macrophages or cell lines such as RAW264.7 or THP-1 has shown that NR4As can regulate their inflammatory profile, lipid uptake and expression of scavenger receptors (Bonta et al., 2006; Hamers et al., 2012; Paoli et al., 2015; Shao et al., 2010). Given that the increased production of IL-12, TNFα and iNOS by stimulated *Nr4a1*^*−/−*^ peritoneal macrophages is sensitive to the NFκB inhibitor Bay-7082 and, as discussed above, given the interplay between NR4As and NFκB signalling, it is likely through the inhibition of this pathway that NR4As modulate the macrophage response and have a positive impact on atherosclerosis (Hanna et al., 2012). However, ChIP data also shows that NR4As can bind to, and regulate the transcription of genes encoding for proteins involved in inflammation or macrophage function such as MERTK and arginase (Dehn and Thorp, 2018; Mahajan et al., 2015). Whether or not this is strictly related to the NFκB pathway is unclear.

One study, where the authors grafted cells from NR4A1 or NR4A3-deficient mice into irradiated *Ldlr*^*−/−*^ recipients, reported no differences in atherosclerotic plaque when compared to wild-type donor cells (Chao et al., 2013). In addition, in the same study, the response of LPS-stimulated peritoneal macrophages from NR4A1 or NR4A3-deficient mice was like the response of wild-type cells in terms of *Il6*, *Tnf*, *Nos2* or *Il12b* gene expression, suggesting no increase in the inflammatory response. Factors such as differences in microbiota, genomic background or techniques were put forward to explain these contradictory results (Chao et al., 2013; Koenis et al., 2018). Nevertheless, most of the data shows that NR4As limit the inflammatory response of macrophages.

### 4.2 The metabolic impact of NR4A1 on macrophages and inflammation

In other tissues, NR4As are known to regulate metabolic processes, and this could contribute to the role they have in inflammation (Pearen and Muscat, 2010). Indeed, in RAW264.7 macrophages overexpressing NR4A1 or not, integrated ChIP-seq and RNA-seq data showed that NR4A1 directly bound and regulated the expression of several metabolic pathways, including genes of the TCA cycle (Koenis et al., 2018). Upon activation, *in vitro* polarized classically activated murine bone marrow-derived macrophages (or M1 macrophages) undergo metabolic reprogramming, generally increasing their dependence on glycolysis for energy requirements, disrupting the TCA cycle at the step catalyzed by isocitrate dehydrogenase, leading to accumulation of succinate (Jha et al., 2015; Nonnenmacher and Hiller, 2018). Succinate is a pro-inflammatory metabolite that drives HIF-1α activity, IL-1β production and reactive oxygen species (ROS) production (Mills et al., 2016; Tannahill et al., 2013). In NR4A1-deficient *in vitro* murine bone marrow-derived macrophages and in RAW264.7 cells knocked-down for *Nr4a1*, all these elements of metabolic reprogramming are affected, resulting in succinate accumulation and in a pro-inflammatory state that can be reverted by chemical inhibition of succinate dehydrogenase (Koenis et al., 2018). *Ldlr*^*−/−*^ mice receiving a bone marrow transplant with NR4A1-deficient cells had increased plasma succinate, inflammatory cytokines and HIF-1α expression in the atherosclerotic lesion, in agreement with the role of NR4A1 in regulating metabolic processes in macrophages and for a role of this metabolic regulation in inflammatory processes (Koenis et al., 2018). The anti-inflammatory roles played by NR4A molecules are thus not entirely dependent on the regulation of the NFκB pathway.

Protective and anti-inflammatory effects of NR4A1 in macrophages have been linked to chronic kidney disease, myocardial infarction, murine lupus and colitis confirming that targeting the activity of this family of receptors could be an interesting therapeutic strategy (Hamers et al., 2015; Hilgendorf et al., 2014; Ipseiz et al., 2014; Westbrook et al., 2014). Experimentally, several molecules have been successfully used. While some such as 6-mercaptopurine, which induces NR4A expression, are not specific to a single NR4A (Huang et al., 2016; Wansa and Muscat, 2005), others seem to exert their function on only one member of the family, suggesting that specific targeting would be possible. This is the case for PDPNA discussed above, which does bind all three NR4A molecules, but because p38α was not found to interact with NR4A2 or NR4A3, it specifically inhibits the interaction between NR4A1 and p38α (Li et al., 2015). Similarly, cytosporone B increased the transcription and transactivational activity of NR4A1 with minimal effect on NR4A2 and NR4A3 (Egarnes et al., 2017; Zhan et al., 2008). In the treatment of diseases with an important macrophage-driven inflammatory component, it is therefore conceivable to target several or a single member of the NR4A family, as needed. However, in some cases, redundancy of these transcription factors may require that NR4A1, NR4A2 and NR4A3 be all targeted for full therapeutic effect.

## 5 Neutrophils and mast cells

The role of NR4As in other myeloid cells such as neutrophils and mast cells is far less explored. *In vitro* generated mouse bone-marrow mast cells and mast cells isolated from human PBMCs respond to activation with bacteria, bacterial products or IgE cross-linking by upregulating NR4As, NR4A3 being the most induced of the three family members (Lundequist et al., 2011). This upregulation is sensitive to NFAT and PKC inhibition (particularly with Gö6976), but the biological significance of the induction of NR4As in mast cells requires further investigation (Lundequist et al., 2011). It is known, however, that bone marrow mast cells from NR4A3-deficient mice produce less MCP-1, TNFα, IL-6 and IL-13 in response to IgE-crosslinking, but produce more of the mast cell protease mMCP-6, suggesting that NR4A3 regulates mast cell function (Garcia-Faroldi et al., 2014). In neutrophils, NR4A3 and NR4A2 were shown to be downstream effectors of PKA for positive regulation of homeostasis and survival (Prince et al., 2017). It could mean that NR4A2 and NR4A3 would be valuable pharmacological targets for disorders where neutrophil accumulation and survival are important features (McCracken and Allen, 2014). Because neutrophils and mast cells must be sensitive to their environment and highly reactive as a first line of defense of the immune system, it would seem probable that immediate early genes such as NR4As have a yet undetermined role in these cells. This is possible given their mRNA expression patterns, that show a link between expression of *Nr4a* and the activation or tissue distribution of neutrophils and mast cells (Dwyer et al., 2016; Ericson et al., 2014; Heng et al., 2008).

## 6 Myeloid malignancies

Myeloid malignancies are clonal expansions of hematopoietic stem cells or progenitor cells. They include acute myeloid leukemia (AML) as well as slow-growing chronic malignancies, several of which can progress to AML (Arber et al., 2016). Chronic malignancies include myeloproliferative neoplasms (MPN), characterized by the proliferation of myeloid lineages found mostly in the spleen and bone marrow, myelodysplastic syndromes (MDS), characterized by ineffective hematopoiesis and abnormal myeloid morphology, as well as MDS/MPNs that have both myelodysplastic and myeloproliferative features. AML is the rapid expansion of abnormally differentiated hematopoietic precursors, myeloblasts, which are found largely in the bone marrow and in the blood. AML is highly heterogeneous, and this heterogeneity represents a significant challenge towards developing treatments. Finding common targetable molecular mechanisms across several AML subtypes to eradicate the disease remains an important goal.

### 6.1 Role of NR4As in AML and other myeloid malignancies

The role of NR4A family members in the development of myeloid malignancies was initially uncovered in a study evaluating the impact of NR4A1 and NR4A3 deficiency in murine myeloid homeostasis (Mullican et al., 2007). Double-deficient mice had an expansion of the HSC compartment and increased infiltration of immature myeloid blasts in the bone marrow, spleen, and peripheral blood. *Nr4a1*^*−/−*^*Nr4a3*^*−/−*^ mice died within 2–4 weeks of birth from this AML-like disease and transplant of bone marrow lacking both *Nr4a1* and *Nr4a3* into irradiated recipients transferred the disease (Mullican et al., 2007). Partial gene reduction also had consequences on hematologic abnormality, as *Nr4a1*^*−/−*^*Nr4a3*^*+/−*^ or *Nr4a1*^+/−^*Nr4a3*^*−/−*^ hypoallelic mice developed chronic myeloid malignancy resembling MDS/MPN. In some cases, this progressed to AML (Ramirez-Herrick et al., 2011). NR4A1 and NR4A3 are therefore redundant tumor suppressors of AML and pre-AML malignancies.

The importance of NR4A1 and NR4A3 in human myeloid malignancy development is supported by the fact that expression of *NR4A1* and *NR4A3* is silenced in AML patient samples with distinct cytogenetic abnormalities and their transcription is reduced in patients with MDS, suggesting that actively silencing these genes is an important step in AML development (Majeti et al., 2009; Mullican et al., 2007; Shimizu et al., 2016; Sternberg et al., 2005). To specifically address this question, Boudreaux et al. generated *Nr4a1*^*fl/fl*^*Nr4a3*^*−/−*^ mice which also had a tamoxifen-inducible Cre enzyme, allowing to test whether leukemia could be induced in adult mice by deleting the floxed *Nr4a1* allele. Indeed, deletion of *Nr4a1* in adult mice in the context of *Nr4a3* deficiency led to AML development. Overexpression of NR4A3 in double deficient leukemic bone marrow cells isolated from these mice prior to transplant into recipients was sufficient to rescue 100% mice from disease when compared to recipients that received leukemic cells transduced with an empty vector (Boudreaux et al., 2012). Therefore, sustained suppression of NR4As is required for AML maintenance. Overexpression of either NR4A1 or NR4A3 in human AML cell lines resulted in decreased cell proliferation and cell survival (Boudreaux et al., 2012; Shimizu et al., 2016) and NR4A3 overexpression reduced the clonogenic potential of hematopoietic progenitors from patients with familial platelet disorder/acute myelogenous leukemia (FPD/AML) (Bluteau et al., 2011), a familial thrombocytopenia with a predisposition to AML. Importantly, overexpression of NR4A1 or NR4A3 with mutations within the DNA binding domain (Saijo et al., 2009) failed to suppress AML cell growth and survival (Boudreaux et al., 2012). This suggests that the DNA binding function and not the conversion of BCL2 into the pro-apoptotic BCL2-BH3 in the mitochondria is the likely mechanism by which NR4As modulate their effects.

### 6.2 Molecular events controlled by NR4As in myeloid malignancies

**Fig. 5:**
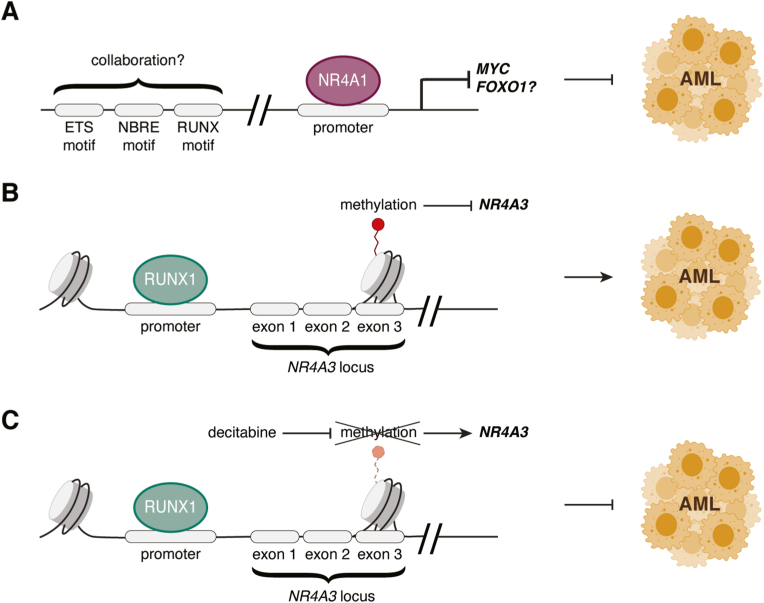
Molecular silencing of NR4As favors *MYC* expression and AML. (A) NR4A binding sites (NBRE motifs) are in proximity to ETS and RUNX motifs, suggesting possible cooperation between these transcription factors. NR4A1 and NR4A3 molecular targets include MYC and FOXO1. Direct repression of MYC is a mechanism by which NR4As inhibit AML development. (B) Repression of NR4A3 in AML can occur directly through RUNX1 inhibition and/or through methylation of an intragenic region around exon 3 of *NR4A3*. (C) Proposed mechanism of action of the hypomethylating agent decitabine on *NR4A3* transcription, which could be beneficial in AML.

Transcriptional analysis of the Kasumi-1 AML cell line as early as 14 h post-transduction with a vector coding for NR4A1 or NR4A3 revealed the redundancy of these proteins in this specific context, with 97% of genes commonly regulated (Boudreaux et al., 2012). Further, these analyses revealed that MYC and its target genes were downregulated following NR4A overexpression and chromatin immunoprecipitation showed that the *Myc* promoter is a direct target of NR4A1 (Fig. 5). Whether NR4A3 is also able to bind to the *Myc* promoter is still unknown. Because MYC is often overexpressed in AML and that this is sufficient to drive AML development in mice, *Myc* transcriptional inhibition could be a mechanism by which NR4As prevent AML development (Alitalo et al., 1985; Hoffman et al., 2002; Luo et al., 2005; Slovak et al., 1994). Importantly, the transcription of 73 genes found to be dysregulated in leukemic stem cells isolated from 9 AML patients (Majeti et al., 2009) was corrected by NR4A1 or NR4A3 overexpression, including *MYC* and *FOXO*1 (Boudreaux et al., 2012). These genes could define an NR4A-regulated signature important for AML development.

Analysis of the genes directly targeted by NR4A1 in Kasumi-1 cells by ChIP-seq was also performed (Duren et al., 2016). This revealed that most of the NR4A1 binding sites were distal from the transcription start site (TSS) and confirmed that *MYC* was a target. Motif analysis revealed that many NR4A1 monomeric NBRE binding sites were close to ETS and RUNX binding sites in both upregulated and downregulated genes, indicating possible cooperation between these molecules to regulate DNA accessibility and gene transcription (Fig. 5). Similar studies for NR4A3 are currently missing.

There are several, not necessarily mutually exclusive ways in which NR4A family members may be silenced in AML. While the promoters of *NR4A1* and *NR4A3* are not targets of hypermethylation (Boudreaux et al., 2019; Shimizu et al., 2016), in NB4, HL60, Kasumi-1, and THP-1 AML cell lines, an intragenic region encompassing exon 3 of *NR4A3* is highly methylated (Fig. 5) (Shimizu et al., 2016). This region, which contains putative regulatory elements, is also more methylated in AML samples from patients. Treatment of AML cell lines with decitabine, a DNA methyltransferase inhibitor, decreased methylation of the targeted region and increased transcription of *NR4A3* (Fig. 5). The possible effects of decitabine on *NR4A1* transcription were not measured in the study, so whether a similar mechanism affects its expression in AML is unclear (Shimizu et al., 2016). Another study showed by ChIP that RNA POL II was recruited at the *NR4A1* and *NR4A3* TSS, but was not found much in intragenic regions, indicating that silencing was the result of poor transcriptional elongation (Boudreaux et al., 2019). Finally, ChIP experiments demonstrated that RUNX1 directly targets *NR4A3* upstream of its transcription start site (Bluteau et al., 2011). Two RUNX1 mutations, R139X and R174Q, found in FPD/AML pedigrees result in decreased active RUNX1 and dramatically reduce NR4A3 activity as measured in a luciferase reporter assay (Bluteau et al., 2011). These results suggest that RUNX1 directly induces NR4A3 and that mutations that lead to a decrease in its activity can contribute to silencing of NR4A3 (Fig. 5).

### 6.3 Pharmacological targeting of NR4As in myeloid malignancies

Because NR4A silencing is a molecular commonality between AML patients with different myeloid malignancies, and because overexpression of NR4A1 or NR4A3 in murine leukemic bone marrow cells or human AML cell lines has desirable effects, it is possibly a valid pharmacological target to treat AML. Using the Connectivity Map (CMap) database (Lamb et al., 2006) that ‘connects’ gene signatures obtained following treatment with bioactive small molecules with cellular signatures resulting from genetic manipulation, Boudreaux *et al.* screened for compounds that could reactivate silenced *NR4A*s and duplicate their transcriptional impact (Boudreaux et al., 2019). Two compounds that scored well in the CMap search were proven to re-induce NR4A1 and NR4A3 expression and inhibit cell growth of the Kasumi-1 cell line: Alprostadil, a prostaglandin, and the ergot alkaloid, dihydroergotamine (DHE). Furthermore, DHE increased RNA POL II binding to intragenic regions, partially reproduced the NR4A genetic program - including repression of MYC - suppressed the viability of cytogenetically distinct AML cell lines and delayed AML progression in mice. In several cancers, including AML, superenhancers are aberrantly activated and drive the expression of oncogenes (Hnisz et al., 2013; Roe et al., 2015). It has been proposed that both DHE and NR4As regulate gene expression by targeting several superenhancers, including the MYC superenhancer (Call et al., 2020). DHE is an FDA-approved drug and its mechanism of action suggests that re-inducing NR4A expression could provide a valid therapeutic target for the treatment of AML.

### 6.4 Role of NR4As in HSC quiescence and homeostasis

The role of NR4A nuclear receptors in HSC homeostasis has been highlighted in other studies (Freire and Conneely, 2018; Land et al., 2015; Sirin et al., 2010). Using a GFP-reporter mouse, Land et al. have shown that *Nr4a1* expression is enriched in quiescent long-term HSCs. Upon transfer into recipients, *Nr4a1*^+^ (GFP^+^) cells gave progeny that was skewed towards the myeloid lineage (Land et al., 2015). Also, NR4A2 overexpression induced HSC quiescence and loss of a single *Nr4a2* allele was sufficient to favor their entry into the cell cycle (Sirin et al., 2010). Tamoxifen-induced acute deletion of *Nr4a1* and *Nr4a3* also resulted in a loss of quiescence in murine HSCs, increased proliferation, and increased DNA damage (Freire and Conneely, 2018). The comparison of transcriptomes of control to co-deleted HSCs revealed that MYC target genes were upregulated in deficient cells, confirming the results obtained in AML cell lines, but also that the gene encoding C/EPBα as well as its associated signature were downregulated in *Nr4a1*^*−/−*^*Nr4a3*^*−/−*^ HSCs (Freire and Conneely, 2018). C/EPBα is a known tumor suppressor mutated in about 10% of AML (Gombart et al., 2002; Pabst et al., 2001) and is a direct target of NR4A1 and NR4A3 (Freire and Conneely, 2018). Thus, NR4A-mediated regulation of HSC quiescence could be via their induction of C/EPBα-driven cell cycle regulation (Porse et al., 2005). Alternatively, ChIP-seq experiments in the Kasumi-1 cell line and ChIP-qPCR in human CD34^+^ bone marrow cells have shown that NR4A1 can bind to NFκB DNA binding sites upstream of regions coding for cytokines such as IL-6, TNFα and IL-1β, possibly competing and limiting the activity of NFκB (Freire and Conneely, 2018). This interpretation is supported by the fact that co-transfection of a luciferase reporter with NFκB DNA responsive elements with a NR4A3-encoding vector resulted in an 80% decrease in reported NFκB activity. Therefore, HSCs deficient for NR4A1 and NR4A3, would acquire an inflammatory profile through overactive NFκB and undergo stress-induced proliferation (Freire and Conneely, 2018; Zhao et al., 2014). Decreased NR4A1 and NR4A3 expression in HSCs could result in their untimely exit from quiescence into a proliferative state favorable to the accumulation of further mutations and progression to leukemia. This would likely be distinct from the role NR4As play in the maintenance of a leukemic state, as discussed above.

From HSC quiescence to leukemic maintenance, there is therefore growing understanding of the molecular importance of NR4As in the biology of myeloid malignancies. Further efforts put towards reactivation of these molecules in AML clones with sufficient specificity to avoid undesired effects of overexpression in other cell types will be essential for this treatment strategy to move to the clinic.

## 7 Concluding remarks

The NR4A family has been linked to several pathologies and whether it would be to treat malignancies, modulate the inflammatory response, harness the unique functions of Ly6C^lo^ monocytes or tune DC-based vaccination strategies (Table 1), there are sufficient arguments to explore the feasibility of targeting NR4As in the clinic. While it may prove difficult to target them pharmacologically given their homology, some molecules such as PDNPA have a degree of specificity that could be exploited. Otherwise, it appears that NR4As sometimes have context-dependent roles that are distinct from the other members of the family, which could also be part of a therapeutic approach. For these reasons, continued research on the common and distinct roles of NR4A1, NR4A2 and NR4A3 in myeloid cells is still required if we wish to fully exploit their clinical potential.

**Table 1:** Roles of NR4As in pathologies.

Pathology	NR4A	Mechanism	Reference
Protective in multiple mouse metastatic tumor models	NR4A1	Induces differentiation into Ly6C^lo^ monocytes that remove unwanted cells	Hanna et al. (2015)
Protective in AML	NR4A1+NR4A3	Inhibit MYC (and probably regulate several more genes) that prevent AML development	(Mullican et al., 2007; Majeti et al., 2009; Sternberg et al., 2005; Shimizu et al., 2016; Boudreaux et al., 2012; Duren et al., 2016)
Protective in Alzheimer's models	NR4A1	Induces differentiation into Ly6C^lo^ monocytes that remove unwanted cells	Michaud et al. (2013)
Detrimental in lung transplants	NR4A1	Donor-derived Ly6C^lo^ monocytes recruit neutrophils that cause tissue damage and primary graft dysfunction	Zheng et al. (2017)
Protective in Parkinson's disease	NR4A2	Prevents reduction of dopaminergic neurons by restraining microglia inflammatory response	(Zetterström et al., 1997; Le et al., 2003; Saijo et al., 2009)
Protective in murine multiple sclerosis	NR4A1	Restrains NE production by macrophages	Shaked et al. (2015)
Protective in sepsis	NR4A1	Restrains inflammatory response of macrophages	Li et al. (2015)
Protective in atherosclerosis	NR4A1, NR4A3	Restrain pro-inflammatory M1 macrophage phenotype	(Bonta et al., 2006; Shao et al., 2010; Hamers et al., 2012; Hanna et al., 2012; Qing et al., 2014; Paoli et al., 2015; Koenis et al., 2018)
Protective in chronic kidney disease	NR4A1	Anti-inflammatory effects	Westbrook et al. (2014)
Protective in myocardial infarction	NR4A1	Anti-inflammatory effects	Hilgendorf et al. (2014)
Protective in murine lupus	NR4A1	Anti-inflammatory effects	Ipseiz et al. (2014)
Protective in murine colitis	NR4A1	Anti-inflammatory effects	Hamers et al. (2015)

## CRediT authorship contribution statement

**Salix Boulet:** Writing – original draft, Writing – review & editing, Conceptualization. **Laure Le Corre:** Writing – review & editing, Visualization. **Livia Odagiu:** Writing – review & editing. **Nathalie Labrecque:** Writing – review & editing, Conceptualization.

## Declaration of competing interest

The authors declare that they have no known competing financial interests or personal relationships that could have appeared to influence the work reported in this paper.
